# Efficient Removal of Fluorine from Leachate of Spent Lithium Iron Phosphate Calcine by Porous Zirconium-Based Adsorbent

**DOI:** 10.3390/ma18235475

**Published:** 2025-12-04

**Authors:** Shengqi Gong, Haijun Huang, Yizheng Wang, Fupeng Liu, Zaoming Chen, Tao Jiang, Ruzhen Peng, Jinliang Wang, Xirong Chen

**Affiliations:** 1 School of Metallurgical Engineering, Jiangxi University of Science and Technology, No. 1958 Kejia Avenue, Ganzhou 341000, China; 2 Jiangxi Ruida New Energy Technology Co., Ltd., No. 8, West Side of Yangguang Avenue, Industrial Park, Yichun 336100, China; 3 Jiangxi Provincial Key Laboratory of High-Performance Steel and Iron Alloy Materials, No. 1958 Kejia Avenue, Ganzhou 341000, China; 4 School of Chemistry and Chemical Engineering, Jiangxi University of Science and Technology, No. 1958 Kejia Avenue, Ganzhou 341000, China

**Keywords:** fluoride ions, spent lithium-ion batteries, zirconium-based adsorbent

## Abstract

During the recycling process of spent lithium-ion batteries (LIBs), there is a large number of fluoride ions in the leaching solution. These fluoride ions not only affect the quality of lithium products, but they also have adverse effects on the environment. Therefore, the efficient and deep removal of the characteristic pollutant fluoride ions is currently a hot topic in the field of recycling spent LIBs. In this study, a porous zirconium-based adsorbent was prepared and its adsorptive properties were characterized. Due to the excellent affinity between zirconium and fluorine, the zirconium-based adsorbent exhibited excellent adsorption performance in the leaching solution of spent lithium iron phosphate (SLFP) batteries. Under the optimal adsorption conditions, the adsorption capacity reached 113.78 mg/g, and it surpassed most commercial adsorbents. The zirconium-based adsorbent followed the Langmuir isotherm model for fluoride adsorption with correlation coefficients consistently exceeding 0.95, and exhibited pseudo-second-order kinetics, demonstrating goodness-of-fit values above 0.998. The negative Gibbs free energy change thermodynamically confirms the spontaneous nature of the adsorption process. The structure of the adsorbent before and after adsorption was characterized, and the adsorption mechanism was elaborated in detail. Furthermore, the influence of the coexistence of different anions on the adsorption of fluoride ions by zirconium-based adsorbent was studied in a real leaching solution from SLFP calcine. This study provides a feasible approach to deep defluoridation for leachate from spent LIBs, and has the advantages of simple operation and high adsorption capacity.

## 1. Introduction

In 2024, new energy vehicle (NEV) stock reached 31.4 million units in China, and the production volume of spent LIBs is expected to increase sharply in the coming years [1,2]. Among them, the lithium iron phosphate (LFP) batteries account for 65% of the share of LIBs [3]. Therefore, the recycling of spent LIBs, especially SLFP batteries, has become a hot topic in recent years. The recycling methods for SLFP batteries mainly include pyrometallurgy, hydrometallurgy, and pyro-hydrometallurgy [4]. Due to the advantages of the pyro-hydrometallurgy method, such as low impurity content, high recovery rate, and easy operation scalability, it has received extensive research. During the recycling process of SLFP batteries, due to the large amount of fluorine-containing substances (such as LiPF_6_) in the electrolyte, they tend to stick together with the black powder [5]. Although some fluorine compounds can be removed during the pyrometallurgy process, the residual fluorine ions in the leaching process hinder adherence to the industrial emission standards.

Critically, fluoride-laden wastewater constitutes a major anthropogenic source of aquatic fluorine pollution [6], and chronic exposure to elevated fluoride levels may lead to irreversible fluorosis and neurodevelopmental disorders [7], indicating the severe threat such contamination poses to global water security and public health. Consequently, research focused on the effective removal of fluoride from wastewater holds significant application prospects and forward-looking importance.

The current treatment of fluorine-containing wastewater has drawn the attention of many scholars, including chemical precipitation [8], electrochemical methods [9], membrane treatment [10], microbial defluoridation [11], adsorption [12], and so on. Among these, chemical precipitation is the most widely applied industrial method for precipitating fluoride ions, but the residual fluoride ions in the solution do not meet the Integrated Wastewater Discharge Standard of China (GB 8978-1996) [13]. The electrochemical fluoride removal process has the advantages of high efficiency and controllability, but high energy consumption and electrode maintenance difficulties limited its application scope. Membrane treatment was employed selective permeability of special membrane materials for defluoridation, has an advantage of high efficiency and environmental friendliness. However, there are also disadvantages such as high membrane costs and the tendency for contamination [14]. Microbial defluoridation as an emerging technology was applied by microbial absorption and oxidation to treat fluoride ions, but it is still at the laboratory research stage due to limited applicability and environmental sensitivity. In recent years, adsorption has become a primary research focus owing to its wide material sources, operational simplicity, rapid kinetics, significant adsorption capacity, simple regeneration, and stable performance [15].

Metal-based adsorbent have been prioritized over biosorbents and mineral-based materials in adsorption research due to their characteristically high specific surface areas and robust chemisorption capabilities toward fluoride ions via surface-active sites [12]. These attributes enable rapid adsorption equilibrium and selective fluoride removal. The main metal-based adsorbent include aluminum-based, iron-based, lanthanum-based, and zirconium-based ones, etc. Among these, zirconium-based materials have been extensively investigated owing to their broad availability, low cost, spontaneous fluoride adsorption, structural stability across regeneration cycles, and environmental compatibility. Specifically, zirconium oxide and zirconium hydroxide, among others, are utilized in the process of removing fluoride from water due to their excellent stability, non-toxicity, and their Lewis acid-base interaction with fluoride ions. The zirconium-based adsorbent have gone through three distinct stages of development, with significant progress made from laboratory research to industrial application. A systematic analysis of this can be found in Text S1 [16,17,18,19,20,21]. The industrial transition toward advanced fluoride removal technologies is not merely progressive but imperative. In conclusion, the progression from laboratory studies to industrial applications underscores zirconium-based materials’ critical role in addressing one of wastewater treatment’s most persistent challenges.

The objective of this study is to develop an economical and scalable zirconium-based adsorbent for deep fluoride removal from the leachate of recycling SLFP batteries. Its core innovation represents the first successful implementation of an adsorbent synthesized exclusively from zirconyl chloride (ZrOCl_2_·8H_2_O) as a sole precursor in authentic industrial wastewater containing extreme sulfate concentrations (>90,000 mg/L SO_4_^2−^), which has never been reported before in prior research. This material achieves high fluoride selectivity in complex systems with Li^+^, Fe^3+^, Al^3+^, and trace PO_4_^3−^ while retaining intrinsic advantages of zirconium oxides (low toxicity, cost-effectiveness, and chemical stability [21]). This approach not only enhances lithium carbonate purity and mitigates environmental fluoride impact but also establishes a scalable deployment framework where conventional methods fail under severe anion interference.

## 2. Experimental

### 2.1. Reagents and Materials

All chemicals used in this study were of analytical grade, including sodium fluoride (NaF, 98.0%) and zirconium oxychloride octahydrate (ZrOCl_2_·8H_2_O, 98.0%) from Macklin, and sodium hydroxide (NaOH), phosphoric acid (H_3_PO_4_), sulfuric acid (H_2_SO_4_), and nitric acid (HNO_3_) provided by Xilong Scientific (Shan tou, China). Deionized water (electrical conductivity < 0.055 μs/cm) was self-made in the laboratory. The leachate was obtained by water leaching of the calcined product (derived from a 1:1 molar ratio mixture of SLFP and sodium sulfate, roasted at 550 °C for 1 h). The leaching was performed at a solid-to-liquid ratio of 10 mL/g in deionized water under continuous stirring at 25 °C for 30 min, which main chemical composition as shown in Table 1. The concentrations of Li^+^, Fe^3+^, Al^3+^, SO_4_^2−^, PO_4_^3−^, and F^−^ in the leaching solution were 1956.0, 4.09, 0.02, 92,424.20, 7.32, and 134.92 mg/L, respectively.

### 2.2. Preparation of Adsorbent

The zirconium oxychloride octahydrate solution with 0.125 mol/L was prepared in the beaker, 0.5 mol/L sulfuric acid solution was introduced dropwise, and the mixture solution was heated to 80 °C for 1 h to promote particle maturation at a speed of 300 rpm in a water bath. The white fine particulate precipitate was collected via centrifugation, followed by triple washing with deionized water to remove residual sulfate ions and soluble impurities. The purified solid was dried at 80 °C for 12 h in an oven to eliminate physisorbed water while preserving porous structure, and subsequently ground using an agate mortar to obtain the porous zirconium-based adsorbent with uniform particle size distribution. The flow chart for the preparation process of zirconium-based adsorbent is shown in Figure S1.

### 2.3. Characterization

The adsorbent was characterized by scanning electron microscopy (SEM, Hitachi Regulus 8100, Tokyo, Japan) coupled with energy-dispersive X-ray spectroscopy (EDS, Oxford Instruments INCA Energy 350, Abingdon, UK) to analyze morphological evolution and elemental distribution pre- and post-adsorption. The specific surface area and pore size distribution of the zirconium-based adsorbent were determined by N_2_ adsorption–desorption isotherms at 78 K using an automated surface area analyzer (Micromeritics ASAP 2460, Norcross, GA, USA). Particle size distribution was analyzed via laser diffraction (Bettersize BT-9300HT/2000E, Dandong, China) with triplicate measurements under dry dispersion mode. The concentration of fluoride ion in the solution was characterized using a fluorine ion concentration meter (PXSJ-216F, Lei-ci Instrumentation, Shanghai, China). Solution pH and fluorine ion concentration were monitored using a PXSJ-261F meter (Leici, Shanghai, China). The ion concentrations in aqueous solutions were tested via inductively coupled plasma–optical emission spectrometry (ICP–OES, Perkin Elmer Optima 7100 DV, Shelton, CT, USA). The adsorbent were identified via X-ray diffraction (XRD, PANalytical X’Pert Pro Powder, Almelo, The Netherlands) using a CuKα radiation source with a 40 kV acceleration potential and current of 40 mA. Phase identification was conducted by matching the diffraction peaks with reference patterns in the ICDD PDF-4+ database (2023 release).

FTIR spectra were recorded using an FTS-135 spectrometer (Bio-Rad, Hercules, CA, USA). Prior to analysis, dried adsorbent samples (before and after adsorption) were thoroughly mixed with spectroscopic-grade potassium bromide (KBr) at a mass ratio of [adsorbent]/[KBr] = 1:100. The mixture was homogenized in an agate mortar and pressed into translucent pellets for FTIR measurement. Background spectra obtained from pure KBr scans were automatically subtracted from the sample spectra. Spectral data were collected in the range of 400–4000 cm^−1^ at a resolution of 4 cm^−1^ with 32 scans per sample to ensure signal-to-noise optimization.

X-ray photoelectron spectroscopy (XPS) measurements were performed on an ESCALAB Xi+ spectrometer (Thermo Fisher Scientific, Waltham, MA, USA) with a monochromatic Al Kα X-ray source (1486.6 eV). The analysis chamber maintained an ultrahigh vacuum of 8 × 10^−10^ Pa. Survey scans (0–1100 eV, passing energy 100 eV, step size 1 eV) and high-resolution regional scans (passing energy 20 eV, step size 0.05 eV, dwell time 40–50 ms) were conducted to characterize elemental composition and chemical states. Operating parameters included an X-ray voltage of 12.5 kV and filament current of 16 mA, with signal averaging applied based on sample conductivity. For depth profiling, argon ion sputtering (1.5 mm spot size, 3000 eV acceleration voltage) was selectively employed. All binding energies were charge-referenced to adventitious carbon (C 1 s at 284.8 eV). Spectral deconvolution utilized a 70% Gaussian/30% Lorentzian line shape in CasaXPS (v2.3.25), following standardized protocols for metal oxide adsorbent.

### 2.4. Adsorption Experiments

#### 2.4.1. Adsorption of Fluoride Ions

A fluoride-containing simulated solution was prepared by dissolving sodium fluoride (NaF, 98.0%) in deionized water, and leachate is derived from water leaching solution (SLFP calcination leachate). The effects of the removal fluoride ion and adsorption capacity were studied in detail at predetermined condition: temperature (25–65 °C), reaction time (0–90 min), adsorbent dosage (1.0–2.0 g/L)and pH (1–13). Meanwhile co-existing ion effects (phosphate [PO_4_^3−^] and sulfate [SO_4_^2−^]) were studied in detail. The fluoride equilibrium adsorption capacity ($q_{e}$, mg/g) of the adsorbent was quantified using the following Equation (1) [22]:

(1) $$q_{e}=\frac{\left(C_{0}-C_{e}\right)V}{m}$$

where $C_{0}$ denotes the initial fluoride concentration (mg/L) in the solution prior to adsorption; $C_{e}$ represents the equilibrium fluoride concentration (mg/L) after reaching adsorption equilibrium; V is the total solution volume (L); m is the mass of the zirconium-based adsorbent (g).

#### 2.4.2. Adsorption Isothermal and Adsorption Kinetics Processes

The removal of the fluoride adsorption isotherm was conducted using a zirconium-based adsorbent three times at various temperatures (25–45 °C). A total of 0.5 g of the adsorbent was added to 50–300 mg/L fluoride solutions in the beakers (500 mL) in a water bath, which was shaken at pH = 7.0 ± 0.1 with a stirring speed of 300 rpm for 8 h. The supernatant was collected to measure the equilibrium fluoride concentration (Cₑ).

The adsorption kinetics experiments were conducted at pH = 7.0 ± 0.1 with a stirring speed of 300 rpm, with the amount of adsorbent (1 g/L), it was added to different concentration solutions (100, 150, and 340 mg/L) in the range of 5 to 180 min, respectively, and the residual fluoride concentration (Cₜ) was determined through ion-selective electrode analysis.

## 3. Results and Discussion

### 3.1. Characterization of Zirconium-Based Adsorbent

**Figure 1:**
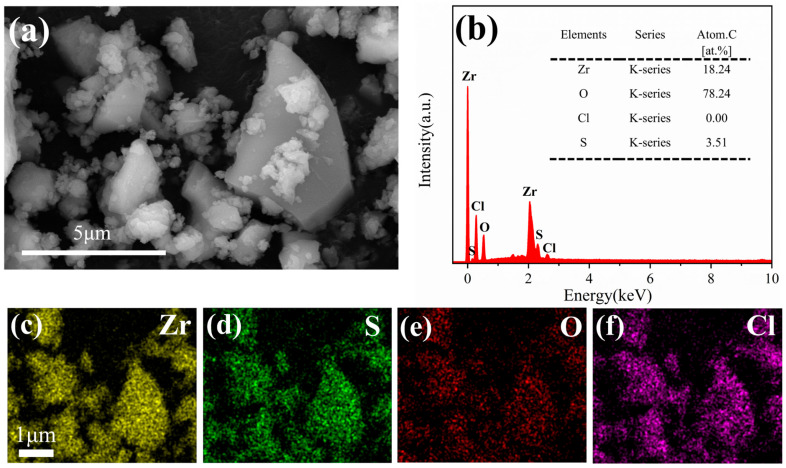

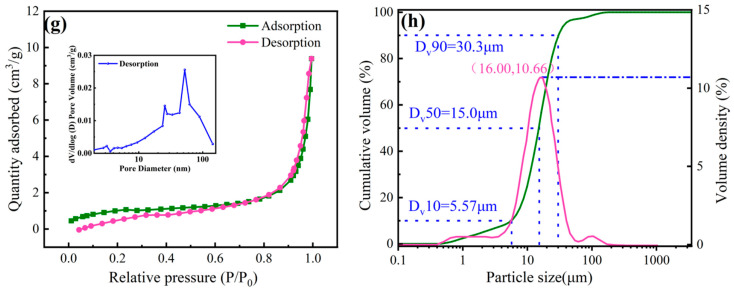
(**a**) SEM images of the surface of the zirconium-based adsorbent; (**b**) pre-adsorption EDS survey spectrum; (**c**–**f**) EDS elemental mappings; (**g**) N_2_ adsorption–desorption isotherms and pore size distribution; (**h**) particle size distribution.

Figure 1a shows that the zirconium-based adsorbent consists of irregular polyhedral particles with an agglomerated morphology, and the observed irregular particle shapes and lack of well-defined crystalline facets suggest the material possesses an amorphous structure [23]. Figure 1b–f revealed that zirconium (Zr) exhibited the highest relative signal intensity among all detected elements, with its spatial distribution pattern closely correlated with sulfur (S) and oxygen (O) across the examined regions. This homogeneous Zr-S-O co-localization strongly suggests the predominant presence of hydrated zirconium hydroxy sulfate (Zr_2_(OH)_6_SO_4_·3H_2_O) as the primary phase, the detected trace chloride ions likely originate from adsorbed counter-ions. These compositional characteristics are consistent with the structural features of similar zirconium-based adsorbent reported by Ma, Yue et al. [24].

As shown in Figure 1g, the N_2_ adsorption–desorption isotherms and pore size distribution profiles of the zirconium-based adsorbent were characterized, the isotherm conforms to a Type IV pattern [25], characteristic of mesoporous solids [26]. At low relative pressures (*p/p*_0_ < 0.1), gradual monolayer adsorption was occurred within slit-shaped mesopores, and a pronounced hysteresis loop (H3-type) emerged at *p/p*_0_ > 0.8, which present interconnected mesopores through capillary condensation in mesochannels. However, a sharp adsorption surge at *p/p*_0_ > 0.9 reflects multilayer adsorption and pore filling in wider mesopores [27], and pore size analysis demonstrates a narrow mesopore distribution centered at 14.92 nm (2–50 nm range, IUPAC definition), and the adsorbent has a hierarchical porous architecture was further explained. It should be noted that the relatively low BET surface area of this zirconium-based adsorbent is likely due to the unfavorable interactions between surface hydroxyl groups [28,29] and N_2_ molecules possessing a quadrupole moment [30], resulting in suppressed adsorption, combined with the absence of a nanoscale porous support carrier, as the material was solely synthesized from a single zirconium oxide compound via a simple hydrothermal method. But compared to other adsorbents, this zirconium-based material shows superior contaminant uptake due to ordered mesochannels facilitating rapid mass transfer [31] and sulfate-functionalized pore walls, enabling selective ion exchange [32].

As shown in Figure 1h, the adsorbent has a polydisperse particle size distribution, with a Sauter mean diameter (*D* [3,2]) of 7.70 μm, volume-weighted mean diameter (*D* [4,3]) of 18.5 μm, median particle size (*D*_v_50) of 15.0 μm, and 90th percentile diameter (*D*_v_90) of 30.3 μm. This multimodal distribution ensures optimal packing density and surface accessibility for adsorption applications, the specific key structural parameters of the zirconium-based adsorbent are listed in Table 2.

### 3.2. Selective Removal Fluoride Ions

#### 3.2.1. The Effect of Initial pH

**Figure 2:**
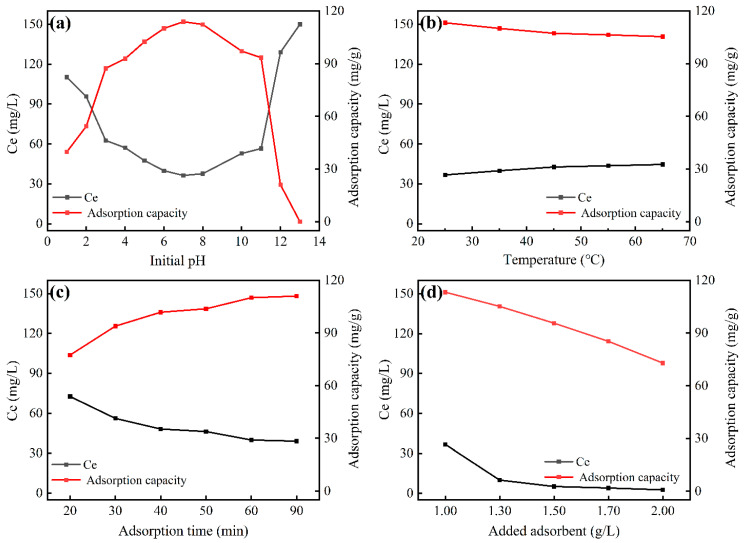
(**a**) Effect of initial pH on fluoride adsorption; (**b**) effect of temperature on fluoride adsorption; (**c**) effect of adsorption time on fluoride adsorption; (**d**) optimum adsorbent dosage.

To investigate the effect of pH on fluoride adsorption, batch adsorption experiments were conducted across an initial pH range of 1–13. As shown in Figure 2a, the adsorption capacity exhibited strong pH-dependence, though the adsorbent maintained broad pH adaptability, adsorption capacity significantly declined under strongly acidic (pH < 3) and alkaline (pH > 11) conditions. This is because when the pH < 3, the main form of fluorine in the solution is HF, even when the pH < 2, the fluoride present in the solution in ionic form can be almost negligible, leading to a decrease in the affinity of fluoride to the surface of the adsorbent [33,34]. Furthermore, under alkaline conditions, the hydroxide ions and fluoride ions in the solution may compete for the active sites, resulting in a significant decrease in the adsorption capacity [33,34,35]. Maximum adsorption capacity (113.78 mg/g) was achieved at pH = 7.0. Since the unadjusted NaF solution (natural pH = 5.93) already showed high adsorption capacity (110.07 mg/g)—only marginally lower than the optimal pH performance—subsequent experiments proceeded without pH adjustment.

#### 3.2.2. The Effect of Temperature

Batch adsorption experiments were conducted across a temperature range of 25–65 °C. As shown in Figure 2b, the zirconium-based adsorbent exhibited minor temperature dependence, with adsorption capacity gradually decreasing as temperature increased, with only 6.97% reduction in fluoride uptake (from 113.78 to 105.39 mg/g) over the 40 °C range. This trend indicates that fluoride adsorption involves electrostatic interactions, where elevated temperatures disrupt the surface potential of the material, thereby reducing adsorption efficiency. Consequently, the optimal adsorption capacity was achieved at 25 °C, which was maintained as the standard temperature for subsequent experiments.

#### 3.2.3. The Effect of Adsorption Time

To investigate the effect of adsorption time on fluoride adsorption, batch experiments were conducted over a duration range of 20–90 min. As shown in Figure 2c, the adsorption rate initially increased and then maintained a high level with prolonged reaction time. This trend is attributed to the progressive occupation of active sites on the material surface [36,37]. The adsorption process gradually reached saturation at 90 min, indicating complete utilization of the zirconium-based adsorbent’s capacity for fluoride ions.

#### 3.2.4. The Effect of Adsorbent Dosage

Fluoride removal to meet the Integrated Wastewater Discharge Standard of China (GB 8978-1996) [13] requires reducing fluoride concentration below 15 mg/L. As shown in Figure 2d, the adsorption capacity gradually decreases with increasing adsorbent dosage, attributable to reduced collision frequency between fluoride ions and active sites [38] on the material surface at lower concentrations. The optimal dosage of this zirconium-based adsorbent is 1.3 g/L for an initial fluoride concentration of 150 mg/L, achieving a stable capacity of 80 mg/g, demonstrating its industrial application potential. Furthermore, increasing the adsorbent dosage effectively reduces the residual fluoride concentration to below 5 mg/L, highlighting its superior deep defluoridation performance.

#### 3.2.5. The Effect on Real Leachate of SLFP Calcine

**Figure 3:**
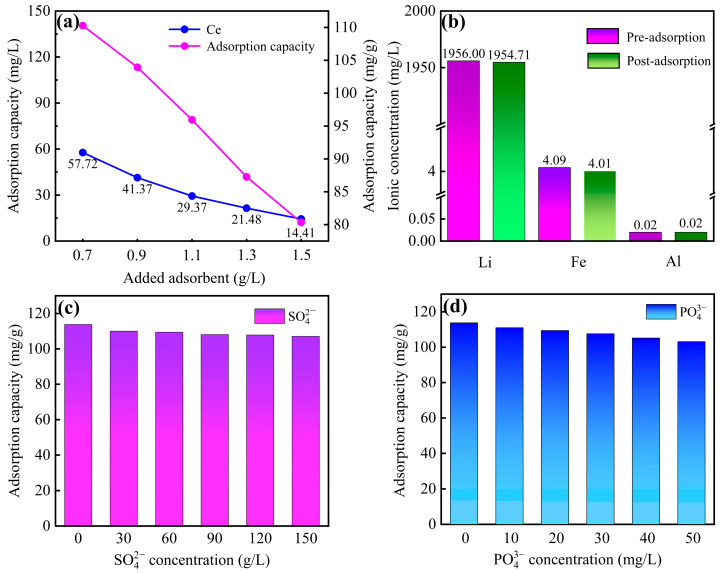
(**a**) Adsorbent dosage versus residual fluoride concentration in real leachate of SLFP calcine; (**b**) variations in metallic element concentrations (Li, Fe, Al) pre- and post-adsorption; (**c**,**d**) the effect of coexisting anions on fluoride adsorption performance of the adsorbent.

Adsorption experiments were carried out using leachate from the water leaching process of SLFP calcine (its composition and ion concentration are shown in Table 1). The changes in metal ion concentrations before and after adsorption were analyzed using an inductively coupled plasma optical emission spectrometer (ICP-OES), and the influence of the amount of adsorbent added on the adsorption effect was investigated. As illustrated in Figure 3a, for the real leachate with an initial fluoride concentration of 134.92 mg/L, a dosage of 1.5 g/L of the zirconium-based adsorbent reduced the fluoride concentration below the Integrated Wastewater Discharge Standard of China (GB 8978-1996). At this dosage, the equilibrium adsorption capacity remained above 80 mg/g, retaining 70.32% of its optimal capacity, demonstrating robust adsorption performance. As shown in Figure 3b, further analysis of metal ion concentrations (Li, Fe, and Al) in the leachate before and after adsorption confirmed negligible interference from coexisting metal ions, reaffirming the selective affinity of the zirconium-based adsorbent for fluoride ions.

#### 3.2.6. Effect of Coexisting Anions

Based on the anion concentrations identified in the leachate of SLFP calcine (Table 1), systematic concentration gradients were established to investigate the interference effects of coexisting anions (SO_4_^2−^ and PO_4_^3−^) on adsorption performance, with results illustrated in Figure 3c,d. As evidenced by the experimental data, coexisting anions at varying concentrations exhibited differential inhibitory effects on the fluoride adsorption performance of the zirconium-based adsorbent, with the suppression intensity following the order: phosphate (PO_4_^3−^) > sulfate (SO_4_^2−^). This trend primarily stems from the preferential formation of inner-sphere complexes between phosphate ions and the adsorbent’s surface hydroxyl groups. Notably, even at low phosphate concentrations (e.g., <50 mg/L), the adsorbent maintained stable fluoride removal efficiency (>90% of its maximum capacity), demonstrating its robustness in multi-anion systems. Therefore, given its wide pH tolerance range, outstanding selectivity, and remarkable stability in multi-anion systems, this adsorbent shows great potential for application in various complex fluoride-rich wastewater streams, including groundwater, drinking water sources [17], surface water bodies [18], and other industrial effluents [21].

### 3.3. Adsorption Isotherm Characteristics and Thermodynamic Analysis

To elucidate the interaction mechanisms between the zirconium-based adsorbent and fluoride ions [39], isothermal adsorption experiments were performed, and three classical thermodynamic models (Langmuir [40] (Equation (S1)), Freundlich (Equation (S2)), and Temkin (Equation (S3))) were plotted [39].

Thermodynamic parameters were calculated from the isotherm model fitting results using the Gibbs free energy equation (Equation (S4)) and Van’t Hoff equation (Equation (S5)) [39,41].

**Figure 4:**
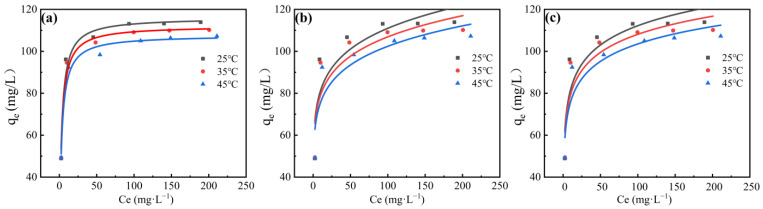
Zirconium-based adsorbent isotherm fitting results: (**a**) Langmuir model; (**b**) Freundlich model; (**c**) Temkin model.

To evaluate the fluoride adsorption performance of the zirconium-based adsorbent and preliminarily understand its adsorption mechanism, three common isotherm models (Langmuir, Freundlich, and Temkin) were employed to fit the adsorption isotherms. The fitting results are presented in Figure 4 with detailed fitting parameters summarized in Table 3.

As clearly demonstrated in Table 3, the adsorption of fluoride ions by the zirconium-based adsorbent exhibited optimal conformity with the Langmuir isotherm model, as evidenced by consistently high correlation coefficients (R^2^ > 0.95) across the tested temperature range (25–65 °C), indicating monolayer chemisorption on homogeneous adsorbent surfaces without intermolecular interactions [39]. The maximum adsorption capacities calculated by the Langmuir model at 25 °C, 35 °C, and 45 °C were 113.3781, 110.1875, and 106.9237 mg/g, respectively, showing excellent agreement with the corresponding experimental values of 113.9, 110.19, and 107.25 mg/g, which exceeds that of most of the reported zirconium-based adsorbent as shown in Table 4 [33]. The Langmuir separation parameter ranged between 0 and 1, confirming favorable adsorption conditions. The Freundlich model parameters 1/n consistently fell within 0.1–0.2, demonstrating highly favorable adsorption with strong affinity between the zirconium-based adsorbent and fluoride ions [39], where adsorption intensity was greater at lower temperatures. This temperature dependence reflects the exothermic nature of spontaneous chemisorption, where increasing temperature reduces saturation adsorption capacity, as evidenced by the 6.57% decrease observed at 45 °C compared to 25 °C.

By investigating temperature effects on the adsorption equilibrium [47], thermodynamic analysis of fluoride adsorption onto the zirconium-based adsorbent was performed. Thermodynamic parameters were derived from the linear relationship between $lnK_{L}$ and 1/T, with the fitting results summarized in Table 5. The Gibbs free energy change (ΔG) was calculated to be −2.83 to −2.43 kJ/mol at 25–45 °C, confirming the spontaneous nature of adsorption, though higher temperatures reduced the thermodynamic favorability. The negative standard enthalpy change (ΔH = −8.77 kJ/mol) reflects the algebraic sum of enthalpy contributions throughout the adsorption process, consistent with the observed decrease in adsorption capacity from 113.78 mg/g at 25 °C to 107.25 mg/g at 55 °C.

According to research by Pengfei Shen et al. on fluoride adsorption by metal oxides, zirconium-based materials follow a “Substitution−Leaching−Deposition (SLD)” mechanism for fluoride ion adsorption [48]. This complex multi-step process means the thermodynamic parameter ΔH cannot definitively determine whether physisorption or chemisorption dominates the fluoride uptake. The negative standard entropy change (ΔS = −19.94 J/(mol·K)) suggests reduced system freedom during adsorption, which aligns with the observed particle size increase in the adsorbent after fluoride uptake. These thermodynamic parameters collectively demonstrate that while fluoride adsorption is spontaneous, elevated temperatures negatively impact the process due to the exothermic nature of adsorption and increased ordering at the solid–liquid interface.

### 3.4. Adsorption Kinetic Characteristics

**Figure 5:**
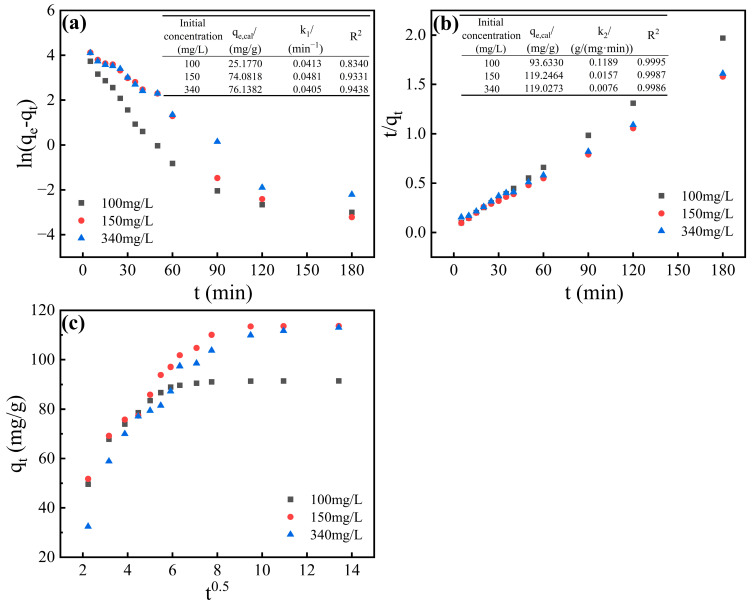
Adsorption kinetics model and fitting parameters: (**a**) the pseudo-first-order (PFO) model; (**b**) pseudo-second-order (PSO) model; (**c**) intra-particle diffusion (IPD) model.

The pseudo-first-order (PFO, Equation (S6)) model [49] for physisorption assessment, the pseudo-second-order (PSO, Equation (S7)) model [50] for chemisorption analysis, and the intra-particle diffusion (IPD, Equation (S8)) model [51] were employed to fit the adsorption kinetics curve. The corresponding fitting results and parameters are comprehensively presented in Figure 5 and Table 6.

As clearly demonstrated in Figure 5, the pseudo-second-order (PSO) model exhibited superior fitting performance compared to the pseudo-first-order (PFO) model across all three fluoride concentrations (100, 150, and 340 mg/L), achieving exceptional fitting accuracy with determination coefficients (R^2^) exceeding 0.998, which confirms that the adsorption mechanism can be featured by the pseudo-second-order model [22]. The theoretically calculated equilibrium capacities from the PSO model (93.63, 119.25, and 119.03 mg/g for 100, 150, and 340 mg/L, respectively) showed excellent agreement with the experimentally obtained values (91.51, 113.71, and 113.14 mg/g), further confirming that the adsorption process was predominantly governed by chemisorption mechanisms [52].

As shown in Figure 5c and Table 6, the intraparticle diffusion model fitting revealed three distinct kinetic stages across all fluoride concentrations [53]: Stage I (0–30 min): Rapid boundary-layer diffusion dominated, evidenced by high rate constants ($K_{i1}$ = 15.11, 14.86, 23.22 mg/(g·min^0.5^) at 100,150,340 mg/L) and significant film resistance (C_1_ = 17.11, 19.64, 23.22 mg/g). The elevated $K_{i1}$ at 340 mg/L (23.22 mg/(g·min^0.5^)) indicates enhanced electrostatic affinity under high fluoride loading Phase II (30–60 min): The pore diffusion control kinetics C_2_ > C_1_, $K_{i2}$ < $K_{i1}$, confirms the greater internal resistance within the particles, indicating that internal diffusion within the particles is the main limiting stage [54]. Notably, the 100 mg/L system achieved exceptional Stage II fitting (R^2^ = 0.9887). Stage III (>90 min): Approached equilibrium with diminishing $K_{i3}$ (0.21, 0.56, 0.77 mg/(g·min^0.5^)) and C_3_ values (88.14, 106.87, 102.90 mg/g) converging near experimental capacities (91.51, 113.71, 113.14 mg/g). As shown in Figure 5c, at different concentrations, the intercept C of the fitting curve of adsorption amount qt and t^0.5^ is not zero, and the fitting curves do not pass through the origin. This indicates that the adsorption process is not solely controlled by internal diffusion of the particles, and chemical adsorption and liquid film diffusion also play a certain role [55].

### 3.5. Mechanisms of Adsorption

#### 3.5.1. SEM and EDS Analysis of the Adsorbent

**Figure 6:**
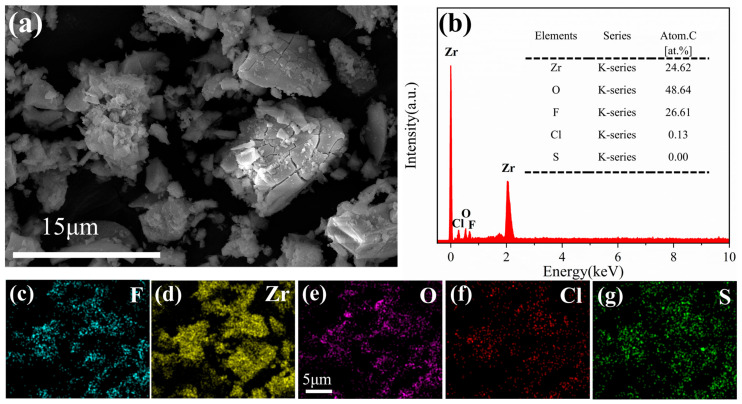
(**a**,**b**) SEM-EDS images and (**c**–**g**) elemental mapping of the post-adsorption adsorbent.

As observed in Figure 6a, the adsorbent exhibits pronounced aggregation into block-like clusters with roughened surfaces and distinct fissures post-adsorption. The evident particle growth aligns with the negative entropy change derived from thermodynamic calculations. Figure 6b reveals co-distribution of fluoride, zirconium, oxygen, and trace amounts of chloride element; However, no sulfur was detected in energy-dispersive X-ray spectroscopy, this result is basically consistent with the analysis result of elemental mapping (Figure 6c–g). Compared to the pre-adsorption state, the atomic percentage of fluorine reached 26.61% after adsorption, further confirming that fluoride ions were adsorbed by the adsorbent.

#### 3.5.2. XRD and FTIR Analysis

**Figure 7:**
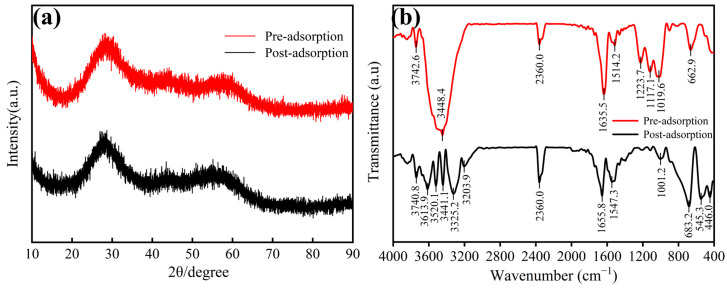
(**a**) X-ray diffraction patterns and (**b**) FTIR spectra of the pre- and post-adsorption.

As shown in Figure 7a, the X-ray diffraction (XRD) patterns of the zirconium-based adsorbent before and after fluoride adsorption display no discernible Bragg diffraction peaks, with a prominent amorphous halo centered at approximately 2θ = 30° (FWHM = 8.5° ± 0.3°, Cu-Kα radiation, λ = 1.5406 Å), confirming its structurally disordered nature. This amorphous architecture endows the material with superior fluoride adsorption performance, achieving a high capacity of 113.28 ± 3.1 mg·g^−1^ at 25 °C and rapid kinetics.

Fourier transform infrared spectroscopy (FTIR) analysis of the zirconium-based adsorbent before and after fluoride adsorption (Figure 7b) revealed characteristic absorption bands. A broad and intense absorption peak centered at 3448.4 cm^−1^ in the range of 3200–3600 cm^−1^ corresponds to the stretching vibration of hydroxyl groups, indicating their presence on the material surface. The sharp peak at 1635.5 cm^−1^ likely originates from crystalline water in the material, which is consistent with the absorption in the 3200–3600 cm^−1^ range. Distinct characteristic peaks at 1223.7 cm^−1^ and 1117.1 cm^−1^ (asymmetric stretching vibrations), along with 662.9 cm^−1^ (asymmetric bending vibration), confirm the presence of SO_4_^2−^ in the zirconium-based material [56], aligning with previous studies by Yue Ma et al. [24].

After fluoride adsorption, the FTIR spectrum showed significant changes: the broad and intense hydroxyl stretching vibration peak at 3441.1 cm^−1^ weakened considerably, suggesting that fluoride ions replaced surface hydroxyl groups (M-OH) to form M-F bonds [57]. The remaining hydroxyl groups, due to the strong electronegativity of fluoride ions, lost their original hydrogen bonding network, reducing intermolecular interactions among hydroxyl groups. This resulted in a sharper peak at 3441.1 cm^−1^ and the appearance of new absorption peaks, confirming the participation of surface hydroxyl groups in fluoride adsorption. Additionally, the intensity of the original peaks at 1223.7 cm^−1^ and 1117.1 cm^−1^ decreased, indicating competitive adsorption between fluoride and sulfate ions for surface sites, which weakened sulfate vibration intensity. Meanwhile, the peak at 662.9 cm^−1^, originally attributed to sulfate asymmetric bending vibration [58], shifted after adsorption, likely due to changes in sulfate coordination caused by strong F^−^-Zr^4+^ interactions.

#### 3.5.3. XPS Analysis

**Figure 8:**
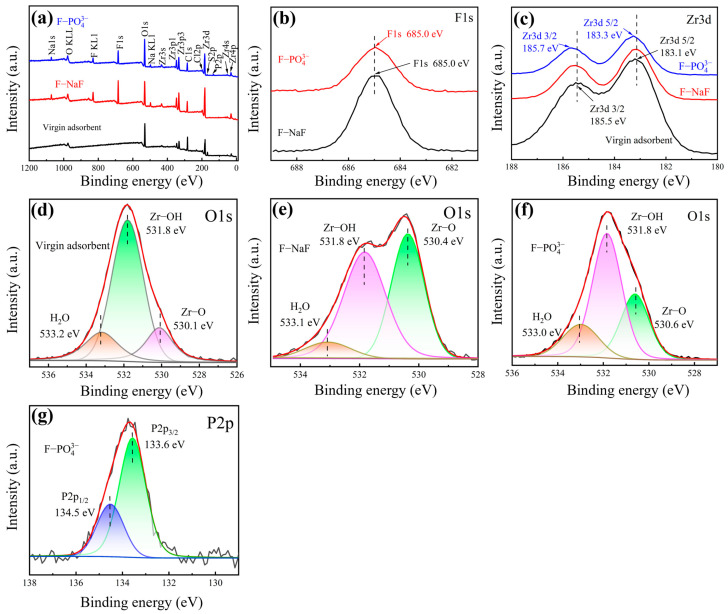
(**a**) Wide scan spectra of the virgin adsorbent, F-loaded adsorbent-NaF and F-loaded adsorbent-PO_4_^3−^; narrow scan spectra of (**b**) F1s, (**c**) Zr3d of pre- and post-adsorption; narrow scan spectra of O1s: (**d**) virgin adsorbent (**e**) F-NaF and (**f**) F-PO_4_^3−^; (**g**) narrow scan spectra of P2p after adsorption in PO_4_^3−^ solution.

XPS was employed to characterize the chemical composition and elemental states of the zirconium-based adsorbent pre- and post-adsorption, with further investigation of phosphate (PO_4_^3−^) interference. The result is shown in Figure 8. Here, F-NaF adsorbent denotes the adsorbent after F^−^ adsorption from a sodium fluoride (NaF) solution, while F^−^-PO_4_^3−^ adsorbent represents the adsorbent after F^−^ adsorption from leachate of SLFP calcine (containing 7.32 mg/L PO_4_^3−^ and lithium sulfate). Figure 8a shows the XPS wide scan spectra of the adsorbent before and after F^−^ adsorption, indicating that O and Zr elements existed in all adsorbents before and after adsorption. After adsorption, a new peak of F1s appears in both F-NaF and F-PO_4_^3−^ adsorbent, demonstrating the adsorption of fluoride by the adsorbent. This confirmed the material’s effectiveness in removing fluoride ions from authentic SLFP calcination leachate. Simultaneously, a new but faint P2p peak was detected on the surface of the F-PO_4_^3−^ adsorbent, indicating weak adsorption binding of phosphate ions. Furthermore, as can be seen in Figure 8a as well, the S2p peaks disappeared after adsorption, indicating that the sulfate ions underwent ion exchange during the adsorption process. The narrow scan spectra of F1s showed that the F1s peak for both F-NaF and F-PO_4_^3−^ adsorbent appeared at 685.0 eV, as shown in Figure 8b.

As shown in Figure 8b, the narrow scan spectra of F1s revealed a peak at 685.0 eV for both F-NaF and F-PO_4_^3−^ adsorbent, closely matching ZrF_4_ (685.1 eV) and differing from NaF (684.5 eV) [59], confirming F^−^ chemically bonded to Zr without NaF deposition (consistent with XRD in Figure 7a). The narrow scan spectra of Zr3d (Figure 8c) showed a binding energy shift after adsorption, suggesting the formation of new zirconium species, potentially including ZrF_4_, ZrO_2_F_5_, or Zr(OH)_2_F_5_ [60]. This shift occurs due to stronger binding energy when zirconium bonds with fluorine. These results confirmed effective F^−^ adsorption.

The narrow scan spectra of O1s pre- and post-adsorption can be divided into three peaks, as shown in Figure 8d–f. The binding energies of the three O1s peaks of the virgin adsorbent are 530.1, 531.8 and 533.2 eV, which correspond to metal oxide (Zr-O), hydroxyl groups bonded to metal (Zr-OH), and H_2_O, respectively [42]. After adsorption in NaF solution and PO_4_^3−^-containing solution, the positions of the three peaks all shifted. For the F-NaF adsorbent, the relative peak area of Zr-O increased from 18.07% to 42.38%, while the relative peak area of Zr-OH decreased from 64.58% to 49.88%, as shown in Figure 8e. The decrease in M-OH indicates that the surface hydroxyl groups of the adsorbent are obviously superseded in the adsorption [61]. For the F-PO_4_^3−^ adsorbent, the relative peak area of Zr-O increased from 18.07% to 28.49%, while the relative peak area of Zr-OH decreased from 64.58% to 55.14%, indicating that the presence of PO_4_^3−^ had a certain impact on F^−^ adsorption, as shown in Figure 8f and Table 7. As shown in Figure 8g, the P2p binding energy peak at 133.6 eV for the F-PO_4_^3−^ adsorbent indicates a small amount of adsorbed PO_4_^3−^, corresponding to the analysis results from the wide scan spectra.

Therefore, the above spectroscopic analysis indicates that the adsorption process of fluoride ions by this zirconium-based adsorbent mainly involves the substitution of surface hydroxyl groups to form M-F bonds, as well as ion exchange involving hydroxyl groups and sulfate ions.

### 3.6. Conclusions and Discussion

In this study, a zirconium-based adsorbent with high efficiency and selectivity was successfully developed for the deep removal of fluorine from the leachate of SLFP calcine. The following conclusions could be drawn:

(1) Elemental analysis determined the molecular formula of the adsorbent as Zr_2_(OH)_6_SO_4_·3H_2_O. N_2_ adsorption–desorption isotherms and pore size distribution confirmed its mesoporous structure, which facilitates fluoride adsorption.
(2) Experimental results showed that the maximum fluoride adsorption capacity of the zirconium-based adsorbent reached 113.78 mg/g at 25 °C and pH = 7.0.
(3) Adsorption isotherm studies indicated that the Langmuir model best described the monolayer chemisorption of fluoride on the adsorbent.
(4) Thermodynamic analysis revealed that fluoride adsorption was a spontaneous and exothermic process.
(5) Kinetic studies demonstrated that the pseudo-second-order model effectively described the adsorption process, suggesting chemical reaction rate control. The adsorbent achieved adsorption equilibrium within 90 min, exhibiting superior fluoride removal efficiency compared to most commercial adsorbents.
(6) The 26.61 atomic% fluorine content in post-adsorption EDS spectra and the emergence of new F1s binding energy in XPS analysis provide direct evidence supporting fluoride adsorption behavior on the zirconium-based adsorbent.
(7) The adsorption mechanism of fluoride ions by this zirconium-based adsorbent mainly involves the substitution of surface hydroxyl groups, ion exchange, and electrostatic adsorption.

However, in order to better explain the pH-dependent adsorption trend, the relationship between the surface zeta potential of the material and the pH value still needs to be further verified. Furthermore, it is necessary to investigate the regeneration and reuse of this zircon-based adsorbent, as this is of crucial importance for industrial applications.

## Figures and Tables

**Table 1:** Ionic concentration of leachate of SLFP calcine in the lithium extraction process (mg/L).

Ionics	F^−^	SO_4_^2−^	Al^3+^	Li^+^	PO_4_^3−^	Fe^3+^	pH
Contents	134.92	92,424.20	0.02	1956.0	7.32	4.09	7.36

**Table 2:** Physicochemical properties (BET surface area, pore size, and particle size distribution) of zirconium-based adsorbent.

Thermophysical Properties	BET Surface Area (m^2^/g)	Micropore Area (m^2^/g)	Micropore Volume (cm^3^/g)	Average Pore Size (nm)	*D* [4,3] (μm)
Zirconium-based adsorbent	3.8892	2.0087	0.000649	14.9227	18.5

**Table 3:** Zirconium-based adsorbent adsorption isotherm fitting parameters.

T (°C)	Langmuir Model			Freundlich Model			Temkin Model		
	$q_{m}$ (mg/g)	$K_{L}$ (L/mg)	R^2^	$K_{F}$ (L/mg)	1/n	R^2^	$K_{T}$	b	R^2^
25	113.3781	0.4373	0.9640	59.6701	0.1348	0.7234	48.3987	187.3361	0.7898
35	110.1875	0.4309	0.9770	58.7578	0.1300	0.7295	59.5049	205.8674	0.7971
45	105.9237	0.4189	0.9776	55.9845	0.1311	0.7702	56.6582	220.7052	0.8332

**Table 4:** Representative emerging zirconium-based adsorbent.

Absorbent Name	pH	BET Specific Surface Area (m^2^/g)	Maximum Adsorption Capacity (mg/g)	Adsorption Isotherm
Y-Zr-Al [42]	7.0	25	31.00	Langmuir
MOF-801 [43]	-	522	17.33	Langmuir
Zr-MCGO [44]	4–8	-	8.84	Koble-Corrigan
La-UiO-66-(COOH)_2_ [33]	3–9	80.249	57.23	Langmuir
Ui-N@PIM-W and Ui-S@PIM-W [45]	2–10	867 and 441	38.74	-
CN-Zr composite material [38]	1–11	87.77	145.34	Langmuir
BTCA-Zr [46]	3–10	-	86	Langmuir
This work	7.0		113.78	Langmuir

**Table 5:** Thermodynamic parameters.

T (°C)	ΔG/(kJ/mol)	ΔH/(kJ/mol)	ΔS/(J/(mol·K))
25	−2.8297	−8.7657	−19.9443
35	−2.6070		
45	−2.4318		

**Table 6:** Intraparticle diffusion model fitting parameters.

Initial Concentration (mg/L)	Stage I (Boundary Layer Diffusion)			Stage II (Pore Diffusion Dominance)			Stage III (Adsorption Equilibrium)		
	$K_{i1}$	C_1_	R^2^	$K_{i2}$	C_2_	R^2^	$K_{i3}$	C_3_	R^2^
100	15.1124	17.1061	0.9185	7.5224	45.1335	0.9887	0.2081	88.1417	0.5757
150	14.8612	19.6391	0.9359	9.4991	38.8417	0.9536	0.5614	106.8706	0.3776
340	23.2163	23.2163	0.9520	7.5139	43.2012	0.9283	0.7650	102.8994	0.8942

**Table 7:** Distribution of Zr-O, Zr-OH, and H_2_O on the adsorbent.

Sample	Peak	Binding Energy/eV	FWHM/eV	Area	Percent/%
Virgin adsorbent	Zr-O	530.1	1.69	38,391.81	18.07
	Zr-OH	531.8	1.96	137,211.27	64.58
	H_2_O	533.2	2.08	36,848.82	17.34
F-NaF	Zr-O	530.3	1.31	30,638.59	42.38
	Zr-OH	531.8	1.67	36,065.36	49.88
	H_2_O	533.1	1.82	5597.37	7.74
F-PO_4_^3−^	Zr-O	530.6	1.47	24,621.10	28.49
	Zr-OH	531.8	1.52	47,655.95	55.14
	H_2_O	533.0	1.84	14,143.47	16.37

## Data Availability

The original contributions presented in this study are included in the article/Supplementary Materials. Further inquiries can be directed to the corresponding authors.

## Supplementary Materials

The following supporting information can be downloaded at: https://www.mdpi.com/article/10.3390/ma18235475/s1. Text S1: The three crucial stages of zirconium-based adsorbent from laboratory research to industrial application. Figure S1: The flow chart for the preparation process of zirconium-based adsorbent. Equations (S1)–(S3): Three classical thermodynamic models. Equations (S4) and (S5): Gibbs free energy equation and Van’t Hoff equation. Equations (S6)–(S8):Three adsorption kinetics models.
