# Supplementary material for: Efficient Removal of Fluorine from Leachate of Spent Lithium Iron Phosphate Calcine by Porous Zirconium-Based Adsorbent
Source: Materials (Basel). 2025 Dec 4;18(23):5475. doi: 10.3390/ma18235475 (PMC12692901; doi:10.3390/ma18235475)
Supplement: Supplementary file 1 [file materials-18-05475-s001.zip › materials-3984803-supplementary.pdf]

SUPPLEMENTARY MATERIAL

Efficient Removal of Fluorine from Leachate of Spent Lithium Iron Phosphate Calcine by Porous Zirconium-Based Adsorbent

Shengqi Gong[1], Haijun Huang[2], Yizheng Wang[1], Fupeng Liu[1,3], Zaoming Chen[*1,3], Tao Jiang[1,3], Ruzhen Peng[*1,3], Jinliang Wang[1,3], Xirong Chen[4]

[1]School of Metallurgical Engineering, Jiangxi University of Science and Technology, No. 1958 Kejia Avenue, Ganzhou, 341000, China.
[2]Jiangxi Ruida New Energy Technology Co., Ltd., No. 8, West Side of Yangguang Avenue, Industrial Park, Yichun, 336100, China.
[3]Jiangxi Provincial Key Laboratory of High-Performance Steel and Iron Alloy Materials, No. 1958 Kejia Avenue, Ganzhou, 341000, China.
[4]School of Chemistry and Chemical Engineering, Jiangxi University of Science and Technology, No. 1958 Kejia Avenue, Ganzhou, 341000, China.
**Corresponding authors.Email addresses: chenzaoming@126.com (Z.C.);9120170001@jxust.edu.cn(R.P.)*

Submitted to **Materials**

October 2025

This file includes supplementary equations:

-6 Pages

-1 text

-1 figure

-8 equations

Table of contents

Text S1 The three crucial stages of zirconium-based adsorbents from laboratory research to industrial application ... 3

Figure S1. The flow chart for the preparation process of zirconium-based adsorbents. ... 4

Equation S1. Langmuir model (monolayer adsorption): ... 4

Equation S2. Freundlich model (heterogeneous surface): ... 4

Equation S3. Temkin model (adsorbate-adsorbate interaction): ... 4

Equation S4. Gibbs free energy equation: ... 4

Equation S5. Van't Hoff equation: ... 4

EquationS6. Pseudo-first-order kinetic model: ... 4

EquationS7. Pseudo-second-order kinetic model: ... 4

EquationS8. Intra-particle diffusion model: ... 5

References ... 5

Text S1 The three crucial stages of zirconium-based adsorbents from laboratory research to industrial application

In the initial phase, researchers focused primarily on simulated fluoride solutions to establish fundamental understanding. The pioneering work of Thakkar et al. (2015) exemplifies this stage, where they successfully immobilized ZrO_2 on microalgal frustules to create a bio-templated composite [1]. While demonstrating good regenerative properties, these early materials exhibited significant limitations, including pronounced pH sensitivity (with adsorption capacity dramatically decreasing above pH 8) and relatively low maximum adsorption capacity (11.32 mg/g), highlighting the constraints of bio-template supported zirconium materials. The second phase saw applications expand to low-concentration natural water systems, marking an important transition toward practical implementation. Parashar et al. (2017) made significant contributions by developing polyaniline-ZrO_2 hybrid materials that achieved effective fluoride removal across a broad pH range (3-9) in groundwater and drinking water [2]. However, these systems proved highly susceptible to interference from common coexisting ions like bicarbonate and phosphate, restricting their use to relatively clean water sources. Other notable advances included Yu et al.'s (2018) mesoporous ZrO_2 fibers for surface water treatment and Patel's (2018) environmentally friendly Al-Zr xerogels [3], though each faced distinct challenges ranging from prohibitive production costs to performance instability in variable water conditions, they all provide important references for the industrial application of zirconium-based materials. The current frontier of the third stage is industrial wastewater treatment, where zirconium-based materials are proving particularly valuable for addressing complex, high-concentration fluoride contamination. Industrial wastewater treatment poses distinct technical challenges characterized by: elevated fluoride concentrations, dynamic pH fluctuations and complex coexisting ion matrices. Among these interfering ions, phosphate (PO_4^{3-}) represents a particularly problematic species due to its exceptional coordination affinity with multivalent cations (e.g., Fe^{3+}, Al^{3+}, Zr^{4+}, Ce^{3+}, La^{3+}). This results in the formation of stable cage-like chelates that competitively occupy active adsorption sites, severely compromising fluoride removal efficiency (Cai et al., 2012; He et al., 2020; Li et al., 2025). Consequently, industrially viable adsorbents must simultaneously exhibit: high and stable adsorption capacity, broad pH adaptability, superior fluoride selectivity, robust anti-interference capability. And the recent breakthrough achievements have shown that the application value of zirconium adsorbents in the industrial field is becoming increasingly prominent, for example: Zhang et al. (2022) developed HZO@D201 nanocomposites by incorporating hydrous zirconium oxide nanoparticles into commercial anion exchange resins, enabling industrial-scale regeneration cycles [4]. However, it still cannot overcome the inherent limitations of its hydrated zirconium oxide. Song et al. (2025) synthesized trimetallic Zr-La-Ce MOFs via hydrothermal method, achieving exceptional Langmuir capacity (167.61 mg/g) through rare-earth incorporation [5], thereby establishing a benchmark for polynuclear MOF design in fluoride capture, but the elevated production costs from lanthanum/cerium elements currently impede large-scale industrial implementation. Shen et al. (2025) engineered switchable microchannel architectures within HIPEs to anchor CLEAs-ZrO_2 composites for industrial wastewater treatment [6], advancing precision fabrication techniques – yet beyond cost constraints, critical gaps persist in evaluating co-existing ion interference (e.g., PO_4^{3-}/SO_4^{2-}/Cl^-) within authentic complex effluents.

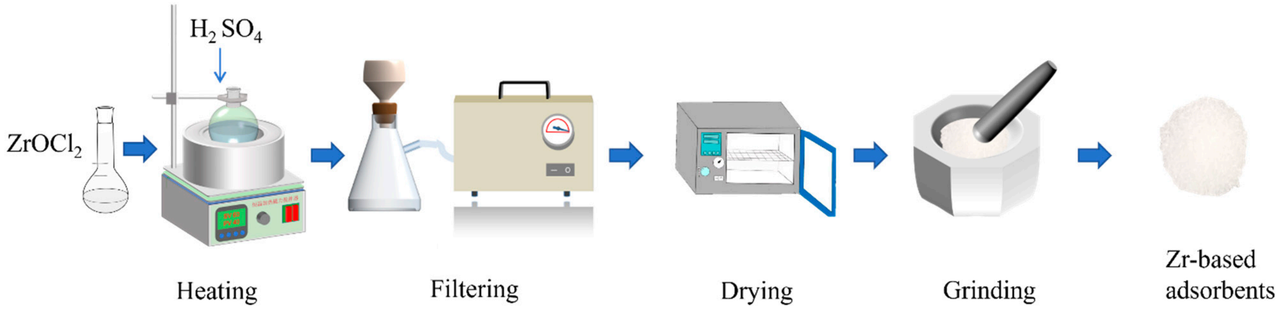

Figure S1. The flow chart for the preparation process of zirconium-based adsorbents.

Equation S1. Langmuir model (monolayer adsorption)[7]:

$$\frac{C_e}{q_e} = \frac{1}{q_m K_L} + \frac{C_e}{q_m} \tag{1}$$

Equation S2. Freundlich model (heterogeneous surface)[8]:

$$\ln q_e = \ln K_F + \frac{1}{n} \ln C_e \tag{2}$$

Equation S3. Temkin model (adsorbate-adsorbate interaction)[8]:

$$q_e = \frac{RT}{b} \ln K_T + \frac{RT}{b} \ln C_e \tag{3}$$

where q_m denotes the theoretical maximum adsorption capacity (mg/g), while q_e reflects the equilibrium adsorption capacity (mg/g). C_e corresponds to the equilibrium fluoride concentration in the solution (mg/L). In the Langmuir model, K_L describes the affinity constant (L/mg), whereas the Freundlich model employs K_F as the adsorption constant (L/mg) and n as the dimensionless heterogeneity factor, where larger values indicate stronger affinity. Thermodynamic analyses involve R, the universal gas constant (8.314 J/mol·K), and T, the absolute temperature (K). The Temkin model utilizes b for the adsorption heat coefficient (J/mol) and K_T for the equilibrium binding constant.

Equation S4. Gibbs free energy equation[8]:

$$\Delta G = -RT \ln K_d \tag{4}$$

Equation S5. Van't Hoff equation[8]:

$$\ln K_d = -\frac{\Delta H}{R} \cdot \frac{1}{T} + \frac{\Delta S}{R} \tag{5}$$

Where ΔG quantifies the standard Gibbs free energy change (kJ/mol). ΔH measures the standard enthalpy change (kJ/mol). ΔS denotes the standard entropy change (J/(mol·K)). Where K_d is the equilibrium constant, defined as $\frac{q_e}{C_e}$ [9].

EquationS6. Pseudo-first-order kinetic model[10]:

$$\ln(q_e - q_t) = \ln q_e - k_1 t \tag{6}$$

EquationS7. Pseudo-second-order kinetic model[11]:

$$\frac{t}{q_t} = \frac{1}{k_2 q_e^2} + \frac{t}{q_e} \tag{7}$$

EquationS8. Intra-particle diffusion model[12]:

$$q_t = k_{ip}t^{0.5} + C \quad (8)$$

where t refers to the contact time (min), q_t measures the adsorption capacity at time t (mg/g). k_1 quantifies the pseudo-first-order (PFO) rate constant (min^{-1}), reflecting physisorption-dominated processes. k_2 describes the pseudo-second-order (PSO) rate constant(mg/(g·$min^{0.5}$)), indicative of chemisorption mechanisms. k_{ip} evaluates the intra-particle diffusion (IPD) rate constant(mg/(g·$min^{0.5}$)), representing pore diffusion efficiency. C defines the boundary layer thickness parameter (mg/g), where C > 0 confirms film diffusion dominance.

References

1. Thakkar, M.; Wu, Z.; Wei, L.; Mitra, S. Water defluoridation using a nanostructured diatom–ZrO_2 composite synthesized from algal Biomass. J. Colloid Interface Sci. **2015**, *450*, 239-245. https://doi:10.1016/j.jcis.2015.03.017.
2. Parashar, K.; Ballav, N.; Debnath, S.; Pillay, K.; Maity, A. Hydrous ZrO_2 decorated polyaniline nanofibres: Synthesis, characterization and application as an efficient adsorbent for water defluoridation. J. Colloid Interface Sci. **2017**, *508*, 342-358. https://doi.org/10.1016/j.jcis.2017.08.044.
3. Patel, S.B.; Panda, A.P.; Swain, S.K.; Patnaik, T.; Muller, F.; Delpeux-Ouldriane, S.; Duclaux, L.; Dey, R.K. Development of aluminum and zirconium based xerogel for defluoridation of drinking water: Study of material properties, solution kinetics and thermodynamics. J. Environ. Chem. Eng. **2018**, *6*, 6231-6242. https://doi.org/10.1016/j.jece.2018.09.031.
4. Zhang, K.; Wei, X.; Ling, C.; Deng, Z.; Zhang, X. Revisiting regeneration performance and mechanism of anion exchanger-supported nano-hydrated zirconium oxides for cyclic water defluoridation. Sep. Purif. Technol. **2022**, *301*, 121906. https://doi.org/10.1016/j.seppur.2022.121906.
5. Song, J.; Ji, L.; Zhang, Z.; Hu, J.; Li, X.-G.; Ma, J. Trimetallic MOFs (Zr–La–Ce) Adsorbent for Defluoridation with Ultrahigh Selectivity and Performance under a Wide pH Range. ACS Appl. Mater. Interfaces. **2025**, *17*, 35353-35363. https://doi.org/10.1021/acsami.4c23068.
6. Shen, D.; Du, Q.; Wang, P.; Zhou, Y.; Pan, J. Confined synthesis of ultrafine ZrO_2 anchoring composites in switchable and microchannel-like space for fluoride removal. Chem. Eng. J. **2025**, *516*, 164275. https://doi.org/10.1016/j.cej.2025.164275.
7. Langmuir, I. The adsorption of gases on plane suafaces of glass, mica and platinum. J. Am. Chem. Soc. **1918**, *40*, 1361-1403. https://doi.org/10.1021/ja02242a004.
8. Al-Ghouti, M.A.; Da'ana, D.A. Guidelines for the use and interpretation of adsorption isotherm models: A review. J. Hazard. Mater. **2020**, *393*,122383. https://doi.org/10.1016/j.jhazmat.2020.122383.
9. Mechnou, I.; Meskini, S.; Elqars, E.; Ait El Had, M.; Hlaibi, M. Efficient CO_2 capture using a novel Zn-doped activated carbon developed from agricultural liquid biomass: Adsorption study, mechanism and transition state. *Surfaces and Interfaces*. **2024**, *52*. https://doi.org/10.1016/j.surfin.2024.104846.
10. Ho, Y.S.; McKay, G. The kinetics of sorption of divalent metal ions onto sphagnum moss peat. Water Res. **2000**, *34*, 735-742. https://doi.org/10.1016/S0043-1354(99)00232-8.
11. Ho, Y. Review of second-order models for adsorption systems. J. Hazard. Mater. **2006**, *136*, 681-689. https://doi.org/10.1016/j.jhazmat.2005.12.043.
12. Wang, J.; Guo, X. Rethinking of the intraparticle diffusion adsorption kinetics model: Interpretation, solving methods and applications. *Chemosphere*. **2022**, *309*,136732. https://doi.org/10.1016/j.chemosphere.2022.136732.
